# Evaluation of apremilast in chronic pruritus of unknown origin: A proof‐of‐concept, phase 2a, open‐label, single‐arm clinical trial

**DOI:** 10.1002/hsr2.154

**Published:** 2020-04-23

**Authors:** Marie Clark, Fang Wang, Nancy D. Bodet, Brian S. Kim

**Affiliations:** ^1^ School of Medicine Saint Louis University St. Louis Missouri USA; ^2^ Division of Dermatology, Department of Medicine Washington University School of Medicine St. Louis Missouri USA; ^3^ Center for the Study of Itch and Sensory Disorders Washington University School of Medicine St. Louis Missouri USA; ^4^ Department of Anesthesiology Washington University School of Medicine St. Louis Missouri USA; ^5^ Department of Pathology and Immunology Washington University School of Medicine St. Louis Missouri USA

## 1 INTRODUCTION

Chronic itch in the absence of a clear etiology is referred to as chronic pruritus of unknown origin (CPUO).^1^ Although CPUO pathogenesis is poorly defined, it is believed that chronic low‐grade inflammation drives itch in this seting.^2^, ^3^ Apremilast, a systemic PDE‐4 inhibitor, is currently approved for a variety of inflammatory disorders in the United States. However, the efficacy of apremilast remains unknown in CPUO. Herein, we present data from an early phase 2a, proof‐of‐concept, open‐label study to investigate the efficacy of apremilast in adults with CPUO.

## 2 METHODS

### 2.1 Study design and treatment

This phase 2a, proof‐of‐concept, open‐label, single‐arm study in adult patients with CPUO was conducted in the United States at one site (http://clinicaltrials.gov identifier: NCT03239106). Patients were recruited, screened, consented, and assessed out of a specialty itch clinic at Washington University School of Medicine during the course of routine clinical care. Key inclusion criteria included age ≥18 years, diagnosis of CPUO for ≥6 weeks, Numerical Rating Scale (NRS) itch score of ≥7, failure of topical triamcinolone 0.1% ointment twice daily (BID) for at least 2 weeks, and one of the histopathological features on skin biopsy in Table S1. Key exclusion criteria included chronic pruritus due to a primary dermatologic or other underlying medical disorder, topical treatments within 1 week of baseline, systemic immunomodulating agents within 4 weeks of baseline, and prior treatment with apremilast. The following medications were prohibited during the study: topical and oral steroids, leukotriene inhibitors, calcineurin inhibitors, allergen immunotherapy, phototherapy, tanning beds, live vaccines, and CYP450 inducers.

While there was no formally stated statistically powered a priori hypothesis for this study, the target enrollment of n = 10 subjects was based on the relative uniformity of the disease severity of the population (ie, severe itch only), and on the fact that we have previously observed relevant differences in populations of CPUO patients with only n = 5 to 6 patients per group in response to treatment.^4^, ^5^ Ten patients with CPUO were enrolled and received 16 weeks of treatment with apremilast 30 mg tablet twice daily (BID).

### 2.2 Assessment

The primary endpoint analysis of this study was absolute reduction in 24‐hour and 1‐week NRS itch score at week 16 from baseline in patients who received apremilast 30 mg BID for 16 weeks. We chose 16 weeks as the primary endpoint in light of recent success at this timepoint with agents employed to treat atopic dermatitis.^6^ The key secondary endpoint was absolute reduction in Dermatology Life Quality Index (DLQI) at week 16 from baseline. Safety and tolerability were assessed by monitoring the type, frequency, duration, and severity of adverse events (AEs) throughout the duration of the study by non‐systematic assessment and self‐reporting by patients at each study visit. The NRS itch score is a single‐question assessment tool with a scale of 0 (no itch) to 10 (worst imaginable itch).^7^ Patients reported their worst level of itch over the prior 24‐hour and 1‐week period at each study visit. Change from baseline in DLQI was also measured to assess patient quality of life (QoL) improvement.^8^ Patients were assessed at baseline and weeks 2, 4, 8, 10, 12, and 16 for these endpoints as well as for vital signs including respiratory rate, pulse, blood pressure, and temperature, and a targeted symptom‐directed physical exam was conducted. Laboratory tests were performed at baseline and at week 16, which included a complete blood count and a comprehensive metabolic profile.

### 2.3 Statistics

All patients were included in the intent‐to‐treat efficacy analysis. Given the unexpectedly high dropout rate and inability to draw any systematic conclusions (see below), we performed a last observation carried forward (LOCF) to week 16 analysis with missing data inferred for the 24‐hour and 1‐week NRS itch scores and DLQI score, in a post hoc manner. All efficacy data points are shown at each individual assessment. Differences in NRS and DLQI scores were assessed via Wilcoxon Signed‐Rank non‐parametric tests for non‐normally distributed data. Differences were considered statistically significant if a two‐tailed *P* < .05. Statistical analysis was performed using GraphPad Prism 8.0 software.

### 2.4 Ethical considerations

The protocol was approved by the Washington University in St. Louis Institutional Review Board (Protocol: 201709093) and informed consent was obtained from all patients.

## 3 RESULTS

### 3.1 Patients

Between December of 2017 and October of 2018, 10 patients were enrolled for treatment. The median [interquartile range, IQR] age was 75 [64.5‐77.5] years and 6/10 were female. The median [IQR] baseline 24‐hour and 1‐week NRS itch scores were 9.25 [7.75‐10] and 8 [7‐9.25], respectively. The baseline mean ± SD DLQI score was 14.3 ± 7.94. All patients exhibited a generalized itch pattern including the trunk and upper and lower extremities as assessed by the principal investigator (PI). The baseline demographics of the patients are shown in Table 1.

**TABLE 1:** Patient demographics and baseline clinical characteristics (N = 10)

Characteristic	Number (%), mean ± SD, or median [IQR]
Age (years)	75 [64.5‐77.5]
Female, number (%)	6 (60%)
White, number (%)	9 (90%)
Black, number (%)	1 (10%)
24‐hour NRS itch score	9.25 [7.75‐10]
1‐week NRS itch score	8 [7‐9.25]
DLQI score	14.3 ± 7.94

Abbreviations: DLQI, Dermatology Life Quality Index; IQR, interquartile range; NRS, Numerical Rating Scale.

### 3.2 Efficacy

**FIGURE 1:**
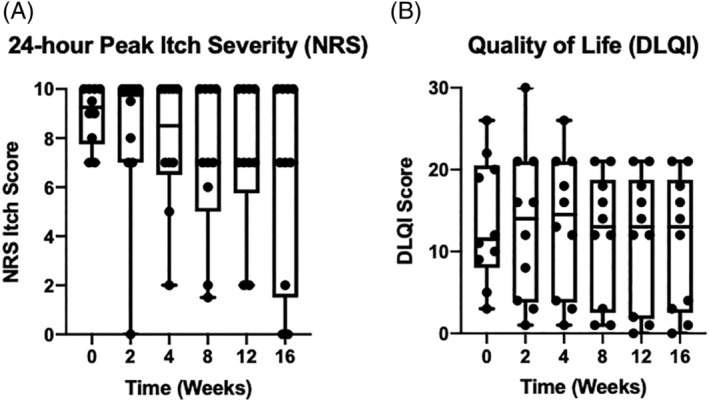
Primary and secondary outcome measures. A, Numerical Rating Scale (NRS) itch scores for patients with CPUO (N = 10) given the PDE‐4 inhibitor, apremilast. Individual patient 24‐hour NRS itch scores are represented by closed circles at baseline (week 0) and weeks 2, 4, 8, 12, and 16. B, Dermatology Life Quality Index (DLQI) scores for a cohort of patients with CPUO (N = 10) given the PDE‐4 inhibitor, apremilast. Individual DLQI scores are represented by closed circles at baseline (week 0), and weeks 2, 4, 8, 12, and 16. Wilcoxon Signed‐Rank nonparametric test was conducted between week 0 and last observation carried forward (LOCF) to test for statistical significance at week 16 for both the NRS Itch Score (*P* = .125) and DLQI Score (*P* = .500). Data are represented as box plots with lines that represent the median value and whiskers which represent range of minimum and maximum values

The data were analyzed in an intent‐to‐treat manner with key primary and secondary endpoints measured as an absolute reduction in NRS itch and DLQI scores, respectively, at week 16 from baseline. In total, 3/10 patients completed the study, which did not allow for meaningful intent‐to‐treat statistical analysis. As an alternative approach, we undertook a post hoc LOCF analysis by carrying forward to week 16. By this analysis, we observed no statistically significant reduction in 24‐hour or 1‐week NRS itch scores at week 16 (Figures 1A and S1). Further, we similarly observed no significant reduction in DLQI at week 16 from baseline (Figure 1B).

Given that 70% of the patients did not complete the study, we sought to examine the reasons for patient dropout. Strikingly, 50% of the patients dropped out due to experiencing an AE. One additional patient opted to discontinue the study due to resolution of itch symptoms, while another desired to use a prohibited medication for itch relief. Two of the three patients who completed the study demonstrated absolute reduction in 24‐hour NRS itch scores from 8 and 9.5 to 0 (Figure 1A). Further, these same subjects also demonstrated a reduction in DLQI score from 26 and 10 to 4 and 0, respectively (Figure 1B). Lastly, one patient completed the study who did not demonstrate a reduction in NRS itch score but a minimal reduction in DLQI score from 5 to 3.

### 3.3 Safety

For this cohort of patients, apremilast was not well tolerated, and several mild to moderate AEs were reported. There were 11 total AEs reported by 5 (50%) patients in the study; all were considered treatment related and resembled AEs reported in the prescribing information for apremilast. The specific AEs and incidences are described in Table 2. Gastrointestinal (GI) dysfunction was reported by 5 (50%) patients, with nausea and diarrhea being the most common. Complaints of nervous system dysfunction were also reported by 2 (20%) patients, which included headaches and presyncope. All AEs were mild to moderate in terms of severity and ceased within 48 hours of stopping the medication. All five patients who experienced AEs dropped out of the study due to the AEs (median [IQR] follow‐up 2 [0‐5] weeks). Notwithstanding this, there were no clinically significant changes as determined by the PI in vital signs, physical exam, or laboratory parameters for any of the patients during the course of the study.

**TABLE 2:** Treatment emergent adverse events (AEs)

AEs	N
Number of patients experiencing an adverse event (% of total)	5 (50%)
Total number of adverse events	11
Gastrointestinal disorders	5
Decreased appetite	1
Nausea	3
Vomiting	1
Diarrhea	3
Nervous system disorders	2
Fatigue	1
Headache	1
Migraine	0
Paresthesia	0
Presyncope	1

*Note:* Values expressed as number (N) unless otherwise indicated.

## 4 DISCUSSION

In the current study, apremilast demonstrated poor tolerability in this population of patients with CPUO, which resulted in high dropout. Because of this, a meaningful intent‐to‐treat statistical analysis was not possible, and we were unable to evaluate both the primary and secondary endpoints of efficacy based on NRS itch and DLQI scores. Therefore, we performed a post‐hoc LOCF analysis, and we did not observe any inferred efficacy. Collectively, we conclude that due to the unexpectedly high rate of AEs in this population, when designing future studies, power analyses would benefit from accounting for a high dropout rate.

Our statistical analysis plan assumed a mean NRS itch score of 8.8 with a SD of 1.1 based on a sampling of patients from our specialty itch clinic. Based on these values and our recent experience in treating small cohorts of patients with CPUO,^4^, ^5^ our target sample size was n = 10. Unfortunately, we did not anticipate such a high dropout rate. Notwithstanding this, two of the three patients who completed the study did demonstrate marked reduction of itch from severe (NRS itch score of 8 and 9.5) to no itch (NRS itch score of 0). This was associated with respective improvement in QoL as measured by the DLQI. However, given that this is not a placebo‐controlled study, we cannot determine whether this is due to a placebo effect or even a direct response to apremilast. As the placebo response in CPUO was recently reported to be surprisingly high in phase 2 clinical trials with other agents such as serlopitant (NCT03841331), studies in this condition will likely require much larger sample sizes than originally anticipated in the design of this study.

## CONFLICT OF INTEREST

Brian S. Kim has served as a consultant to AbbVie, Cara Therapeutics, Concert Pharmaceuticals, Incyte Corporation, Menlo Therapeutics, Pfizer, and Sanofi‐Genzyme; has served on advisory boards for Cara Therapeutics, Boeringher Ingelheim, Celgene Corporation, Kiniksa Pharmaceuticals, Menlo Therapeutics, Regeneron Pharmaceuticals, Sanofi‐Genzyme, and Theravance Biopharma; is a shareholder in Locus Biosciences and Nuogen Pharma; and is founder and chief scientific officer of Nuogen Pharma. The work was funded by Celgene, however, neither Celgene nor any of the listed entities had any role in the data collection, analysis, interpretation, writing of the report, or decision to submit for publication.

## AUTHOR CONTRIBUTIONS

Conceptualization: Marie Clark, Fang Wang, Brian S. Kim

Formal Analysis: Marie Clark, Fang Wang, Brian S. Kim

Funding Acquisition: Brian S. Kim

Investigation: Marie Clark, Fang Wang, Nancy D. Bodet, Brian S. Kim

Methodology: Marie Clark, Fang Wang, Brian S. Kim

Project Administration: Brian S. Kim

Supervision: Brian S. Kim

Writing—review and editing: Marie Clark, Fang Wang, Brian S. Kim

Writing—original draft: Marie Clark

All authors have read and approved the final version of the manuscript. Brian S. Kim had full access to all of the data and takes complete responsibility for the integrity of the data and the accuracy of the data analysis.

## TRANSPARENCY STATEMENT

The corresponding author, Brian S. Kim, affirms that this manuscript is an honest, accurate, and transparent account of the study being reported; that no important aspects of the study have been omitted; that any discrepancies from the study as planned have been explained.

## Supporting information


**Table S1** Skin biopsy features from most pruritic skin sites by histopathology at baseline (H&E and tryptase stain).

**Figure S1** Skin biopsy features from most pruritic skin sites by histopathology at baseline (H&E and tryptase stain).

## Data Availability

Individual de‐identified participant data will be shared immediately following publication with investigators whose proposed use of the data has been approved by an independent review committee identified for this purpose. Proposals should be directed to briankim@wustl.edu. To gain access, data requestors will need to sign a data access agreement. Study protocol will be made available on http://clinicaltrials.gov (NCT03239106).
