# Supplementary material for: Evaluation of apremilast in chronic pruritus of unknown origin: A proof‐of‐concept, phase 2a, open‐label, single‐arm clinical trial
Source: Health Sci Rep. 2020 Apr 23;3(2):e154. doi: 10.1002/hsr2.154 (PMC7178825; doi:10.1002/hsr2.154)
Supplement: Supplementary file 1 — Table S1 Skin biopsy features from most pruritic skin sites by histopathology at baseline (H&E and tryptase stain). [file HSR2-3-e154-s001.docx]

**SUPPLEMENTAL DATA**

**Table S1. Skin biopsy features from most pruritic skin sites by histopathology at baseline (H&E and tryptase stain).**

| **Patient** | **Dermal edema** | **Eosinophils** | **Lymphocytic infiltrate** | **Activated mast cells** | **Spongiosis** |
| --- | --- | --- | --- | --- | --- |
| 1 | X |  | X |  |  |
| 2 |  | X | X |  |  |
| 3 |  | X | X |  | X |
| 4 | X |  |  | X |  |
| 5 | X |  |  | X |  |
| 6 | X |  | X |  |  |
| 7 | X |  |  | X |  |
| 8 | X |  |  | X |  |
| 9 |  | X | X |  | X |
| 10 |  | X | X |  |  |

The presence of one or more of these features is denoted by an “X” mark.
