# Supplementary material for: Evaluation of apremilast in chronic pruritus of unknown origin: A proof‐of‐concept, phase 2a, open‐label, single‐arm clinical trial
Source: Health Sci Rep. 2020 Apr 23;3(2):e154. doi: 10.1002/hsr2.154 (PMC7178825; doi:10.1002/hsr2.154)
Supplement: Supplementary file 2 — Figure S1 Skin biopsy features from most pruritic skin sites by histopathology at baseline (H&E and tryptase stain). [file HSR2-3-e154-s002.docx]

**Supplemental Figure 1.** Numerical Rating Scale (NRS) itch scores for patients with CPUO (N=10) given the PDE-4 inhibitor, apremilast. Individual patient 1-Week NRS itch scores are represented by closed circles at baseline (week 0) and weeks 2, 4, 8, 12, and 16. Wilcoxon Signed-Rank non-parametric test was conducted between week 0 and last observation carried forward (LOCF) to test for statistical significance at Week 16 (P = 0.14). Data are represented as box plots with lines that represent the median value and whiskers which represent range of minimum and maximum values.
